# *Salmonella enterica* Serovar Typhi with CTX-M β-Lactamase, Germany

**DOI:** 10.3201/eid1509.090567

**Published:** 2009-09

**Authors:** Yvonne Pfeifer, Jens Matten, Wolfgang Rabsch

**Affiliations:** *Salmonella enterica* Serovar Typhi with CTX-M β-Lactamase, Germany; Robert Koch Institute, Wernigerode, Germany (Y. Pfeifer, W. Rabsch); MVZ Labor Nord-West GmbH, Nordhorn, Germany (J. Matten)

**Keywords:** Salmonella, CTX-M-15 beta-lactamase, Qnr, typhoid, antimicrobial resistance, bacteria, letter

**To the Editor:** Infection with *Salmonella enterica* serovar Typhi, the causative agent of typhoid fever, is an acute systemic illness with a high proportion of illness and deaths, especially in developing countries. In Europe, *S. enterica* ser. Typhi infections occur among travelers returning from disease-endemic areas. After emergence of multidrug-resistant *S. enterica* ser. Typhi strains that confer resistance to chloramphenicol, trimethoprim, and ampicillin, quinolones have become the primary drugs for treatment (*1*). Here we report the isolation of a CTX-M–producing *S. enterica* ser. Typhi in Germany.

We isolated *S. enterica* ser. Typhi from blood and feces specimens from a 30-year-old Iraqi woman who was admitted to the hospital in Cologne in August 2008. The patient was febrile, dizzy, and had epigastric pain and headache. The symptoms began 2 weeks earlier, after she had returned from a month-long visit to her relatives in Sulaymaniya, the capital of As Sulaymaniyah Governorate in the northeastern Iraqi Kurdistan region. The interview indicated that the same symptoms had developed in other family members in Iraq. The patient was treated successfully with meropenem (1 g 3×/day) for 2 weeks, and no relapse was observed in a follow-up period of 6 months.

The isolated strain was identified as *S. enterica* ser. Typhi with the VITEK2 system (VITEK2 GN-card; bioMérieux, Brussels, Belgium) and by slide agglutination with *Salmonella* antisera (SIFIN, Berlin, Germany) in accordance with the Kauffmann-White scheme. By using Vi-phage typing according to the International Federation for Enteric Phage Typing (L.R. Ward, pers. comm.), the strain was classified as *S*. *enterica* ser. Typhi Vi-phage type E9. Antimicrobial drug susceptibilities were determined according to the guidelines of the Clinical Laboratory Standards Institute with the VITEK2 AST-N021 card and Etest (bioMérieux). The extended-spectrum β-lactamase (ESBL) phenotype was confirmed with a combined disk diffusion test (MASTDISCS ID, Mast Diagnostica GmbH, Germany). PCR and sequence analyses were performed with universal primers for the ESBL genes *bla*_CTX-M,_ *bla*_TEM_, and *bla*_SHV_ as described previously (*2*). Primer CTX-M-F 5′-TTCGTCTCTTCCAGAATAAGG-3′ and primer CTX-M-R 5'-CAGCACTTTTGCCGTCTAAG-3′ were used for sequencing the entire blaCTX-M gene. Investigation of the CTX-M environment was performed with primers IS*26*-F (5′-GCCTGGTAAGCAGAGTTTTTG-3′) and IS26-CTX-R (5′-ACAGCGGCACACTTCCTAAC-3′). The presence of plasmid-mediated quinolone resistance genes (*qnr*) was determined by PCR and sequencing of *qnrB* (*3*), *qnrS* (primer F, 5′-CGGCACCACAACTTTTCAC-3′; primer R, 5′-CAACAATACCCAGTGCTTCG-3′), and *qnrA* (primer F, 5′-ATTTCTCACGCCAGGATTTG-3′; primer R, 5′-CGGCAAAGGTTAGGTCACAG-3′). In addition, the nucleotide sequences of the quinolone resistance-determining regions of the *gyrA*, *gyrB*, *parC,* and *parE* genes were determined as previously described (*4*). Transfer of β-lactam resistance was tested by broth mating assays with a sodium azide–resistant *Escherichia coli* J53 recipient. Selection of transconjugants was performed on Mueller-Hinton agar plates that contained sodium azide (200 μg/mL) and ampicillin (100 μg/mL). We isolated the plasmid DNA of donor and transconjugants using the QIAGEN Plasmid Mini Kit (QIAGEN, Hilden, Germany).

Phenotypically, the strain was resistant to ampicillin, ampicillin/sulbactam, piperacillin, cefotaxime, ceftazidime, cefepime, chloramphenicol, streptomycin, trimethoprim/sulfamethoxazole, azithromycin, and nalidixic acid. A reduced susceptibility to ciprofloxacin was detected (MIC_CIP_ = 1 μg/mL). The isolate was susceptible to imipenem, meropenem, gentamicin, tobramycin, and amikacin. PCR and sequence analyses displayed the presence of *bla*_CTX-M-15_, *bla*_TEM-1_ and the *qnrB2* gene. We found an amino acid substitution in *gyrA* gene (83-Ser→Phe). No mutations were identified in the *gyrB*, *parC*, and *parE* genes. Sequencing of the insertion element (IS)*26*-F/R amplification product showed the location of IS*26 transposase A* gene *(tnpA),* followed by a truncated IS*Ecp1* mobile element upstream of the *bla*_CTX-M-15_ gene. By conjugation, 1 plasmid of ≈50 kbp was successfully transferred into an *E. coli* J53 recipient (Figure). PCR-based replicon typing (*5*) showed an IncN–related plasmid. The *E. coli* J53 transconjugant mediated resistance to ampicillin, cefotaxime, ceftazidime, cefepime, trimethoprim/sulfamethazole, nalidixic acid and showed reduced susceptibility to ciprofloxacin (MIC = 0.5 μg/mL). Also, in the transconjugant, the *bla*_CTX-M-15_ and *qnrB2* genes were identified by PCR.

**Figure:**
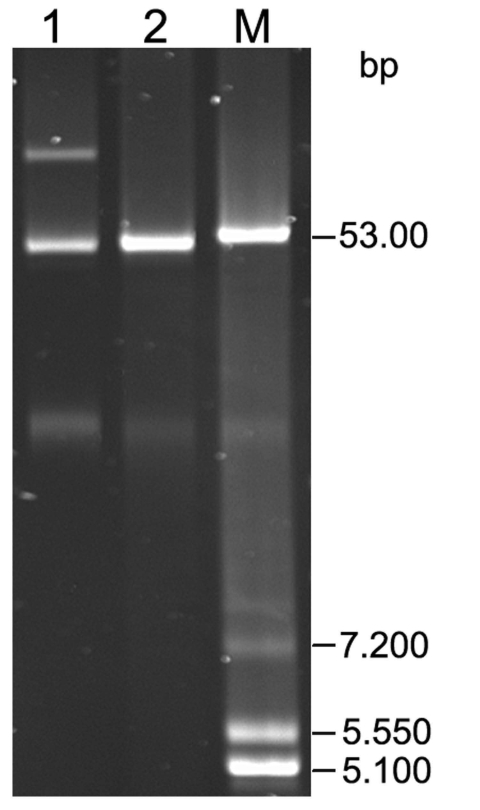
Plasmids isolated from *Salmonella enterica* serovar Typhi and *Escherichia coli* J53 transconjugant. Lane 1, *S. enterica* ser. Typhi 218/08 (*bla*_CTX-M-15_ + *bla*_TEM-1_ + *qnrB2*); lane 2, *E. coli* J53 transconjugant (*bla*_CTX-M-15_ + *qnrB2*); lane M, plasmid marker *E. coli* V517.

ESBL-producing non-Typhi serotypes of *S. enterica* are an increasing problem worldwide. In Europe and Asia, CTX-M-group ESBLs are prevalent in *S. enterica,* and in North America, domestically acquired CTX-M ESBLs were recently identified in *S. enterica* ser. Typhimurium (*6*). In *S. enterica* ser. Typhi, reports of ESBLs have been rare. The CTX-M-15 type that we found has been reported only once previously in *S. enterica* ser. Typhi from Indian patients hospitalized in Kuwait (*7*). In addition to cephalosporin resistance mediated by ESBLs, the reduced susceptibility to quinolones in *S. enterica* is of concern. In the study isolate, this reduced susceptibility was due to a known mutation 83-Ser→Phe in *gyrA* (*8*) and the acquisition of a *qnB2* gene. Plasmid-mediated Qnr determinants have been identified in *S. enterica* of different non-Typhi serovars (*9*), whereas in *S. enterica* ser. Typhi, only mutations in gyrase and topoisomerase genes leading to quinolone resistance had been observed previously (*8*).

In our isolate of *S. enterica* ser. Typhi that contained *bla*_CTX-15_ and *qnrB2,* resistance to cephalosporins as well as the reduced quinolone susceptibility was easily transferable by conjugation into *E. coli.* This occurrence is alarming because the dissemination of such strains with acquired resistances will further limit the therapeutic options for treatment of typhoid fever.
